# Multifocal intraocular lenses and retinal diseases

**DOI:** 10.1007/s00417-020-04603-0

**Published:** 2020-01-18

**Authors:** Andrzej Grzybowski, Piotr Kanclerz, Raimo Tuuminen

**Affiliations:** 1 Department of Ophthalmology, University of Warmia and Mazury, Olsztyn, Poland; 2 Institute for Research in Ophthalmology, Foundation for Ophthalmology Development, Poznan, Poland; 3 Hygeia Clinic, Gdańsk, Poland; 4 Helsinki Retina Research Group, Faculty of Medicine, University of Helsinki, Helsinki, Finland; 5 Department of Ophthalmology, Kymenlaakso Central Hospital, Kotka, Finland

**Keywords:** Age-related macular degeneration, Cataract surgery, Contrast sensitivity, Diabetic retinopathy, Multifocal intraocular lens, Refractive lens exchange

## Abstract

**Purpose:**

Multifocal intraocular lenses (MIOLs) are often discouraged in patients with or at risk of retinal disorders (including diabetic retinopathy, age-related macular degeneration, and epiretinal membranes), as MIOLs are believed to reduce contrast sensitivity (CS). Concerns with MIOLs have also been raised in individuals with visual field defects, fixation instability or eccentric preferred retinal locations. The aim of this study is to review the influence of MIOL on quality of vision in patients with retinal diseases.

**Methods:**

We reviewed the PubMed and Web of Science databases to identify relevant studies using the following keywords: multifocal intraocular lens, cataract surgery, cataract extraction, lens exchange, diabetic retinopathy, age-related macular degeneration, and contrast sensitivity.

**Results:**

Studies evaluating CS in MIOLs present conflicting results: MIOLs either did not influence CS or resulted in worse performance under low-illuminance conditions and higher spatial frequencies when compared to monofocal IOLs. Nevertheless, MIOLs preserved CS levels within the age-matched normal range. Two studies reported that patients with concurrent retinal diseases receiving a MIOL, both unilaterally and bilaterally, reported a significant improvement in visual-related outcomes. Individuals with a monofocal IOL in one eye and a MIOL in the fellow eye reported greater subjective satisfaction with the MIOL.

**Conclusion:**

We were unable to find evidence suggesting that patients with retinal diseases should be advised against MIOLs. Nevertheless, more research is needed to address the aforementioned concerns and to optimize the use of MIOLs in eyes with retinal disease.

**Electronic supplementary material:**

The online version of this article (10.1007/s00417-020-04603-0) contains supplementary material, which is available to authorized users.

## Introduction

Advances in cataract surgery and refractive lens exchange have enabled these techniques to be used to achieve precise and desired refractive outcomes[1]. This has raised patients’ expectations of excellent uncorrected distance visual acuity[2]. However, with monofocal intraocular lenses (IOLs), the desire for freedom from spectacles cannot be completely met. Multifocal intraocular lenses (MIOLs) are used more commonly and to a considerable extent for refractive purposes in non-cataractous eyes [3]. It is believed that as MIOLs decrease contrast sensitivity, so they are advised against in patients with retinal disorders.

MIOLs utilize diffractive or refractive optics in order to produce two or more foci. A diffractive MIOL generates multifocality based on light interference. It incorporates a pattern consisting of a series of annular concentric grooves less than 1 micron in depth, which are engraved around the optical axis on either the front or the back surface of a lens (the echelette technology). With the refractive design, multifocality is achieved with light refraction on the MIOL surfaces based on Snell’s law. The performance of refractive design MIOLs depends on pupil size and MIOL centration.

The increasing popularity of MIOLs underlines the importance of reviewing scientific evidence regarding their suitability in patients with ocular comorbidities to aid preoperative assessment and proper patient selection in MIOL candidates. The aim of this study is to review the influence of MIOL on quality of vision in patients with retinal diseases.

## Materials and methods

The PubMed and Web of Science databases were the main resources used to investigate the medical literature. An extensive search was performed to identify relevant articles concerning “multifocal intraocular lenses” and “retinal diseases” up to June 30, 2018 (Appendix 1). The following keywords were used in various combinations: multifocal intraocular lens, cataract surgery, cataract extraction, lens exchange, diabetic retinopathy, age-related macular degeneration, and contrast sensitivity. The search identified 247 unique articles. Of the studies retrieved by this method, we reviewed all publications in English and abstracts of non-English publications. The reference lists of the articles analyzed were also considered as a potential source of information. We attempted to present all publications that employed MIOLs for patients with retinal diseases. Studies were critically reviewed to create an overview and guidance for further research. No attempts were made to discover unpublished data. In addition to the PubMed and Web of Science searches, selected chapters from relevant textbooks were included.

## Results

### Contrast sensitivity (CS) and age

Various procedures and systems are employed to evaluate contrast sensitivity. The results of these evaluations be affected by differences in methodology, including light conditions, speed of performing the test, or decision-making criteria. In 1978, Arden and Jacobson claimed that contrast sensitivity is independent of age, and since then, several studies have sought an association between contrast sensitivity and age [4]. Studies analyzing the relationship between CS and age are presented in Table 1. It might be concluded that contrast sensitivity decreases with age and that the greatest decrease is observed at higher spatial frequencies. Age and cataract are independently associated with this decline, and posterior subcapsular cataract affects CS in the highest degree. Lower CS was also reported in females and in Chinese, when compared to other races present in Singapore [5, 6]. Moreover, CS at higher spatial frequencies is lower in myopes compared to emmetropes [5].

**Table 1:** Studies analyzing the relationship between contrast sensitivity and age in a general population

Study	Subjects (age)	Parameter analyzed	Results
Karatepe et al. 2017 [7]	37 individuals (aged 7–65 years)	CS at 0.5, 1.5, 3.0, 6.0, 12.0, and 24.0 cpd at illumination levels of 0–30 dB (dB)	Increasing age, small pupil diameter, hyperopia, and photopic conditions were associated with lower contrast sensitivity in healthy individuals
Sia et al. 2013 [8]	472 adults aged 35–80 years	CS at 3, 6, 12, and 18 cpd	CS decreases with age at all spatial frequencies and is greatest at highest spatial frequencies. Posterior subcapsular cataract causes reduction at all frequencies, while cortical cataract does not. Nuclear cataract decrease CS at intermediate (12 cpd) and high (18 cpd) frequencies
Hohberger et al. 2007 [9]	61 subjects (categorized in age groups < 30 years, 30–39 years, 40–49 years, 50–59 years, > 60 years)	CS at 1.5, 3, 6, 12, and 18 cpd, under day (85 cd/m^2^) and night (3.0 cd/m^2^) conditions, with and without glare	Contrast sensitivity was significantly reduced under night conditions with glare, whereas glare had less influence under daylight illumination. Regression analyses showed a highly significant influence of age, but the variance of the measurement values is not explained by age alone
Nomura et al. 2003 [10]	2267 subjects (aged 40–79 years)	CS at 1.5, 3, 6, 12, and 18 cpd	The age-related decrease in CS was found at all frequencies, even when adjusted for visual acuity
Nio et al. 2000 [11]	100 subjects (20–69 years of age	CS at 1, 2, 4, 8, and 16 cpd; pupil size 2, 4, and 6 mm; defocus −1 to +2 D	At optimal focus, integrated contrast sensitivity and log CS at 8 cpd showed a significant age-related decline. The log CS at 1 cpd was independent of age
Klein et al. 1996 [6]	5926 individuals (43–84 years of age)	CS measured with a perimeter in the 25° central field	Visual sensitivity was inversely associated with age and was lower in women in each age stratum
Burton et al. 1993 [12]	35 young (aged 17–29 years) and 29 older (aged 60–80 years) subjects	CS at 2, 4, 8, 12, 16, 20, 24, 28, and 32 cpd	Older adults in good eye health exhibited on average a small loss (0.1–0.2 log unit) in contrast sensitivity across the spatial frequency range tested
Nameda et al. 1989 [13]	19 individuals (aged 24–63 years)	CS at 1.5, 2, 3, 4, 5, 6, 7, 8, 9, 10, 15, 20, 30, 40, 50, 60, and 70 cpd	Losses at high spatial frequencies up to 40 years of age. After 40 years of age, there were losses at all spatial frequencies
Tulunay-Keesey et al. 1988 [14]	63 adults (13–67 years of age)	CS at spatial frequencies of 0.5, 1, 2, 3, 4, 6, 8, and 12 cpd and temporal frequencies of 0, 1, 5, and 15 Hz	Sensitivity for low spatial frequencies modulated at 0 to 15 Hz was not affected by age, but a progressive age-related elevation of threshold was found for combinations of high spatial and temporal frequencies
Crassini et al. 1988 [15]	8 young (average age 20.4 years) and 8 elderly (average age 64.4 years) subjects	CS (central an 10 deg temporally) at 0.2, 0.8, 2.0, and 5.0 cpd	Young observers had better contrast sensitivities than older observers but only at higher spatial frequencies (2.0 and 5.0 cpd)
Sloane et al. 1988 [16]	12 young (19–35 years) and 11 older (68–89 years) subjects	CS as a function of target luminance at 0.5, 2, 4, and 8 cpd	When gratings were flickered at 0.5 Hz, functions for older adults were displaced downward on the sensitivity axis across all luminance levels, and the slopes of these functions were steeper than those for younger adults, suggesting that optical mechanisms alone cannot account for the vision loss in older adults.
Higgins et al. 1988 [17]	50 subjects in five age groups (20–29, 30–39, 40–49, 50–59, 60–69 years)	CS at nine spatial frequencies from 0.75 to 16 cpd	Decline in sensitivity with age at all spatial frequencies
Elliot 1987 [18]	16 young (mean age 21.5 ± 2.7 years) and 16 older (mean age 72 ± 4.3 years) subjects	CS at 1, 2, 4, 10.6, and 16.5 cpd	Lower contrast sensitivity at medium (4 cpd) and high (10.6, 16.5 cpd) spatial frequencies in older group
Yates et al. 1987 [19]	103 adults (21–40 years of age)	CS at 1, 2, 4, 8, 12, 16, 20, and 24 cpd	The age-related decrease in CS was found only at 16 cpd
Ross et al. 1985 [20]	17 young (aged 20–30 years) and 53 older (aged 50–87 years) subjects	CS at 0.4, 0.95, 2.88, 6.73, 12.70, and 19.23 cpd	Lower performance in older group compared to younger group at all spatial frequencies In the older group, linear decline in CS with age for medium and high spatial frequencies
Morrison and McGrath 1985 [21]	45 observers (including 4 elderly)	CS 8–40 cpd (10–15 different frequencies within this range)	With increasing age, CS remained steady until the sixth decade when they declined
Kline et al. 1983 [22]	16 young (18–25 years) and 16 old (55–70 years) subjects	CS at 0.5, 2, 4, 6, 8, and 12 cpd	An age-related loss in contrast sensitivity was observed primarily for stimuli of intermediate and high spatial frequency
Owsley et al. 1983 [23]	91 adults aged 19–87 years	CS at 0.5, 1, 2, 4, 8, and 16 cpd	At higher spatial frequencies. Sensitivity decreased with age around 40 to 50 years
McGrath and Morrison 1981 [24]	66 subjects (5–94 years old)	CS at 1, 2, 3, 5, 10, 20, and 25 cpd	With advancing age, there was an overall decrease in contrast sensitivity. The loss of CS was greater for middle range spatial frequencies than for higher spatial frequencies
Sekuler et al. 1980 [25]	25 young (mean age 18.5 ± 0.7 years) and 10 old (73.2 + 3.8 years) individuals	CS at 0.5, 1, 2, 4, 8, and 16 cps flickered at 0.3 or 6 Hz	Older and younger observers did not differ in ability to see targets with fine structure (high spatial frequencies); older observers were only one-third as sensitive to targets with coarse structure (low spatial frequencies) as were younger observers. Older observers were also less able than younger observers to see moving targets
Derefeldt et al. 1979 [26]	10 children (aged 6–10 years), 12 adults (aged 20–40 years), 5 adults (aged 40–60 years), 11 adults (aged 60 years or older)	CS at 0.5, 1.0, 2.0, 4.0, 10, 20, and 40 cpd	No significant difference between young- and middle-aged subjects with regard to contrast sensitivity. For higher ages studied (above 60 years), there was a loss of sensitivity in the middle and high frequency regions
Arden & Jacobson 1978 [4]	50 healthy adults aged 17–64 years, 7 eyes with ocular hypertension, 43 eyes with glaucoma	CS at 0.4, 0.8, 1.6, 3.2, and 6.4 cpd	The variation in the test results with age in normals is only slight.

*cpd* cycles per degree, *CS* contrast sensitivity

Burton et al. [12] suggested that optical rather than neural changes in the visual system contribute to loss of spatial contrast sensitivity at a photopic level in the elderly. This small loss in CS was presumably due to interaction of a laser speckle effect and increased light scattering in the aged eye. On the other hand, Elliot et al. [27] reported that the decrease in contrast sensitivity is due to neural loss. In their study, senile miosis and reduced optical transmission, which are believed to influence CS, were mimicked in younger subjects by reducing retinal illumination and did not result in a decrease in CS. Higgins et al. [17] believe that the high-spatial frequency decline in CS, although it is comparatively small, is too large to be due to changes in lens density related to age. This was confirmed by Owsley et al. [23], who demonstrated that after cataract surgery, elderly patients still have decreased CS at higher frequencies, although they have a crystalline IOL. The decline in visual performance might also be due to spatial integration and difficulty in processing visual information in the presence of noise [28]. Morrison and Jay [29] confirmed with laser interferometry that the optical functions with aging remain unchanged, while neural function significantly declines.

### CS and MIOLs

Studies analyzing CS in MIOLs present conflicting results, as seen in Table 2. Comparing CS outcomes is difficult due to the differences in the tests used and different illumination levels and inconsistencies in the variables reported. One might conclude that CS with MIOLs was lower than with monofocal IOLs in at least some conditions; in some studies, MIOLs performed worse under low-illuminance conditions and at higher spatial frequencies. The outcome regarding CS might depend on the MIOL model and design: diffractive optics influence CS to a lesser degree [30]. However, the quality of evidence is poor. Furthermore, none of the studies presented an association between CS and the quality of vision [31]. CS improved with time and achieved an age normal range of 3–12 months after surgery. Similar results were presented in the meta-analysis by Rosen et al. [32] and in the review by Wang et al. [33]. Thus, doubts remain as to which MIOL design would offer the best visual performance and patient satisfaction in patients with retinal disease.

**Table 2:** Contrast sensitivity in multifocal intraocular lenses

Study	IOLs (number of eyes)	Parameter analyzed	Results
Altermir-Gomez et al. 2019 [34]	Tecnis ZCB00 (AMO, *n* = 44) vs. Tecnis ZMB00 (AMO, *n* = 78)	CS at 3, 6, 12, and 18 cpd (mesopic and photopic)	No difference in CS
Menucci et al. 2018 [35]	PanOptix IQ (Alcon, *n* = 40), AT LISA tri 839MP (Carl Zeiss Meditec, *n* = 40), Tecnis Symfony (AMO, *n =* 40)	CS at 1.5, 3, 6, 12, and 18 cpd (mesopic and photopic)	The Tecnis Symfony MIOLs provided significantly better photopic and mesopic CS outcomes than the other MIOL models
Dyrda et al. 2018 [36]	OptiVis (Aaren Scientific, *n* = 64) vs. AR40E (AMO, *n* = 64) vs. M-Flex (Rayner, *n* = 64) vs. ReZoom (AMO, *n* = 64) vs. ReSTOR (Alcon, *n* = 64)	CS at 1.5, 3, 6, 12, and 18 cpd	Only under mesopic conditions without glare, distance CS with the MIOL was significantly lower than with the monofocal IOL at any tested frequencies
Pedrotti et al. 2018 [30]	Tecnis (AMO, *n* = 30), vs. Tecnis Symfony (AMO, *n* = 55), vs. ReSTOR +2.5 (Alcon, *n* = 50), vs. ReSTOR +3.0 (Alcon, *n* = 50)	CS at 6, 12, and 18 cpd (85 cd/m^2^ at 4 m)	No differences in photopic CS between the monofocal IOL and the EDOF Tecnis Symfony in any spatial frequency. In contrast, all contrast sensitivity values for these two lenses were significantly better than those obtained with both apodized diffractive refractive ReSTOR MIOLs
Pedrotti et al. 2018 [37]	Comfort LS-313 MF 15 (Lentis, *n* = 11) vs. monofocal Tecnis (AMO, *n* = 12)	CS at 3, 6, 12, and 18 cpd	no differences in CS
Maxwell et al. 2017 [38]	Monofocal Acrysof SN60WF (Alcon) vs. Acrysof IQ Restor +2.5	CS at 3, 6, 12, and 18 cpd (photopic); mesopic 1.5, 3.0, 6.0, and 12.0 cpd with and without glare	No relevant differences in binocular CS under photopic or mesopic conditions, with or without glare
Plaza-Puche et al. 2016 [39]	AT LISA tri 839MP (Carl Zeiss Meditec, *n* = 30), FineVision (Physiol, *n* = 30), MPlus-LS313 (Lentis, *n* = 30), AcriLisa 366D (Carl Zeiss Meditec, *n* = 30), ReSTOP SN6AD1 (Alcon, *n* = 30), monofocal spherical Acri.Smart 48S (Carl Zeiss Meditec, *n* = 30)	CS at 1.5, 3, 6, 12, and 18 cpd in low mesopic levels	No differences in CS in low mesopic conditions at 1.5, 3, 6, and 12 cpd but only at 18 cpd. In pair comparison found better values for monofocal than ReSTOR at 18 cpd
Labiris et al. 2015 [40]	multifocal Isert PY60MV (Hoya, *n* = 37) vs. monovision SN60WF (Alcon, *n* = 38)	CS with Pelli-Robson chart	No differences in CS
Gil et al. 2014 [41]	Acrysof ReSTOR SN6AD1 (Alcon, *n* = 35) vs. Acrysof ReSTOR SN60D3 (Alcon, *n* = 36) vs. Tecnis ZMA00 (AMO, *n* = 38) vs. ReZoom NXG (AMO, *n* = 33) vs. Tecnis ZA9003 (AMO, *n* = 38)	distance CS at 3, 6, 12, and 18 cpd (8 levels of contrast), near CS at 1.5, 3, 6, 12, and 18 cpd (8 levels of contrast)	Monofocal better at all spatial frequencies. Diffractive optics and aspheric profiles showed a non-statistically significant trend to perform better in mesopic conditions. Near CS was lower for refractive, distance dominant lens designs, particularly at medium to high spatial frequencies
Tan et al. 2014 [42]	Akreos AO (Bausch & Lomb) vs. ZMA00 (AMO) vs. Tetraflex (Lenstec) (total *n* = 128 eyes)	CS visual acuity was measured at contrast levels: 100%, 25%, 10%, and 5%	No significant differences of the CS visual acuity were present among the three groups at 3 months after surgery
Yamauchi et al. 2013 [43]	Tecnis ZA9003/ZCB00 (AMO) vs. ZMA00/ZMB00 (AMO)	CS with optotype size 0.7, 1, 1.6, 2.5, 4, and 6.3 degree with and without glare	CS (with and without glare) was better in the monofocal group
Ji et al. 2013 [44]	monofocal Acrysof Natural (Alcon, *n* = 27) vs. Acrysof ReSTOR (Alcon, *n* = 24)	CS at 0.7, 1.0, 1.6, 2.5, 4.0, and 6.3 cpd; scotopic (80 cd/m^2^) and mesopic (5 cd/m^2^) conditions	Monofocal presented better CS at all spatial frequencies and conditions
Wilkins et al. 2013 [45]	Tecnis ZM900 (AMO, *n* = 106) vs. monovision Akreos AO (Bausch & Lomb, *n* = 105)	CS with Pelli-Robson chart	Monovision better than multifocal (*p* = 0.009)
Zhao et al. 2010 [46]	Acrysof ReSTOR SA60D3 (Alcon, *n* = 72) vs. Acrysof SA60AT (Alcon, *n* = 89)	CS at 1.5, 3, 6, 12, and 18 cpd	Better CS at 3 cpd in monofocal IOL
Martínez Palmer et al. 2008 [47]	Tecnis Z9000 (AMO, *n* = 48) vs. Tecnis ZM900 (AMO, *n* = 52) vs. ReZoom (AMO, *n* = 64) vs. TwinSet (AcriTec, *n* = 64)	CS at 1.5, 3, 6, 12, and 18 cpd in mesopic and scotopic conditions, with and without glare	Mean contrast sensitivity was better for the monofocal IOL group than for the MIOLs. Patients assigned to TwinSet had less favorable contrast sensitivity scores compared to newer design multifocals
Cillino et al. 2008 [48]	AR40 (AMO, *n* = 15) vs. Array SA40N (AMO, *n* = 16) vs. ReZoom (AMO, *n* = 15) vs. ZM900 (*n* = 16)	CS at 1.5, 3, 6, 12, and 18 cpd	All groups behaved similarly. At 3 cpd, the monofocal IOL (AR40) and diffractive pupil-independent MIOL (ZM900) groups exhibited a higher sensitivity than the refractive MIOL groups (ReZoom and ZM900) (*P* = 0.038)
Harman et al. 2008 [49]	1CU (*n* = 28) vs. Array SA40N (AMO, *n* = 27) vs. Clariflex (AMO, n = 27)	CS with Pelli-Robson chart	CS slightly higher in 1CU than in array at 3 months, No differences at 18 months
Zeng et al. 2007 [50]	Z9001 (AMO, *n* = 40) vs. SA60AT (Alcon, *n* = 45) vs. SA40N (AMO, *n* = 39)	CS at 6, 12, and 18 cpd (85 cd/m^2^ at 4 m) with and without glare	Z9001 showed better CS than SA40AT, while SA60AT better than SA40N (significant at all spatial frequencies)
Sen et al. 2004 [51]	SI-40NB (AMO, *n* = 67) vs. Array SA-40 N (AMO, *n* = 53)	CS at 1.5, 3, 6, 12, and 18 cpd	CS was slightly lower with MIOLs at all spatial frequencies; the difference was not significant and decreased over time
Montés-Micó et al. 2004 [52]	SI-40NB (AMO, *n* = 32) vs. Array SA-40 N (AMO, *n* = 32)	CS at 1.5, 3, 6, 12, and 18 cpd	As low luminances, distance CS for MIOL worse than monofocal IOL for highest spatial frequencies (12 and 18 cpd). Under bright conditions no difference – CS within normal limits
Leyland et al. 2002 [53]	S140 N (AMO) vs. Array SA40NB (AMO) vs. TrueVista 68STUV (Storz)	CS with Pelli-Robson chart at 1 m	Mean binocular contrast sensitivity was 1.74 (SD 0.15) for the monofocal IOL, 1.67 (0.13) for the multifocal, and 1.65 (0.20) for the bifocal (unclear in statistically significant)
Kamlesh et al. 2001 [54]	Progres 3 (Domilens, *n* = 20) vs. Flex 65 (Domilens, *n* = 20)	CS with Pelli-Robson chart at 90 cd/m^2^	Mean CS was lower in patients with MIOLs than those with a monofocal IOL (1.38 vs. 1.56 log units; *p* < 0.001)
Haaskjold et al. 1998 [55]	Diffractive bifocal PMMA 808X (Pharmacia, *n* = 115) vs. monofocal 808D (Pharmacia, *n* = 106)	CS at 1.5, 3, 6, 12, and 18 cpd at distance and near	Bifocal had lower CS than monofocal IOL at all spatial frequencies
Allen et al. 1996 [56]	Diffractive bifocal PMMA 808X (Pharmacia, *n* = 79) vs. monofocal 808D (Pharmacia, *n* = 70)	CS at 1.5, 3, 6, 12, and 18 cpd at three light levels	Differences in CS at all light levels. Particularly in medium light, bifocal group had reduced CS compared with monofocal IOL but still within normal limits
Percival et al. 1993 [57]	PC25 (AMO, *n* = 25) vs. Array (AMO, *n* = 25)	Regan system	Slightly lower CS in MIOLs than monofocal IOLs at all contrast levels, not statistically significant
Steinert et al. 1992 [58]	PC-25NB (AMO, *n* = 30) vs. Array MPC-25NB (AMO, *n* = 32)	Regan system	MIOL worse than monofocal IOL only at 11% contrast level

*CS* contrast sensitivity, *IOL* intraocular lens, *MIOL* multifocal intraocular lens

## Discussion

### Contraindications for MIOLs

Several doubts and contraindications for MIOL implantation currently exist. These include corneal aberrations, particularly coma or cone, which might result in a decreased contrast sensitivity and dysphotopsia. IOL decentration relative to the pupil center leads to an increase in high-order aberrations and diminished objective contrast discrimination. Thus, an asymmetric capsulorrhexis, haptics deformation, or finally IOL subluxation might disqualify a patient from an MIOL. As the IOL pseudoexfoliation syndrome is correlated with zonular instability, centration in these patients is questionable. Patients with a large pupil, postoperative ametropia, and astigmatism or who have developed posterior capsule opacification are at risk of dissatisfaction after MIOL implantation [59, 60]. Multifocals are strictly disadvised in retinitis pigmentosa and Stargardt’s disease, while diabetic retinopathy, age-related macular degeneration, and epiretinal membranes are relative contraindications [61]. In light of this, a thoughtful approach would be recommended in patients with neuro-ophthalmological conditions (e.g., strabismus, Horner’s syndrome) or glaucomatous visual field defects [62, 63].

### Evidence on MIOLs and retinal diseases

Only two studies assessed the visual outcome of MIOLs in patients with concurrent retinal diseases (Table 3). Kamath et al. [64] reported that patients with concurrent eye diseases including age-related macular degeneration, glaucoma, or diabetic retinopathy benefited from the multifocality of an IOL. The MIOL arm of the study comprised 81 eyes (70 patients) and the monofocal arm 52 eyes (41 patients). Distance visual acuities were similar in the monofocal IOL and MIOL groups, while uncorrected near visual acuity (UNVA) was significantly better in the MIOL group. Patients who had a monofocal IOL in one eye and a MIOL (Array, Abbott Medical Optics Santa Ana, CA) in the fellow eye reported greater subjective satisfaction with the MIOL; however, 3 of these 11 patients had more advanced pathology in the eye with the monofocal IOL, making it difficult to interpret the significance of this finding. However, no clear definition of “more advanced pathology” was given by the authors. Our study demonstrated that the results are divergent, even when they only include normal eyes without concurrent retinal diseases. Nevertheless, one might question whether the presence of eye diseases should be a decisive reason to categorically advise against MIOLs.

**Table 3:** Studies assessed visual outcomes of MIOL implantation in patients with concurrent retinal diseases

Study	IOL	Diseases	Conclusion
Gayton et al. [63]	Acrysof Restor targeting −2.0D (Alcon)	Dry age-related macular degeneration or macular degeneration associated with disciform scarring.	In cataractous eyes with age-related macular degeneration, replacing the crystalline lens with this myopia-targeted multifocal intraocular lens improved or maintained near vision without severely compromising distance vision
Kamath et al. [62]	Multifocal array (AMO) vs. monofocal SI-40NB0 (AMO)	Age-related macular degeneration Glaucoma Ocular Hypertension Diabetic Retinopathy Others	The IOLs presented similar distance visual outcomes; however, a proportion of patients benefited from the IOLs’ multifocality

Gayton et al. [65] proposed implantation of Acrysof Restor (Alcon, Fort Worth, TX) targeting − 2.0D in eyes with age-related macular degeneration and corrected distance visual acuity (CDVA) of 20/50 or worse. This approach is particularly interesting as it provided an uncorrected near + 5.2D addition. The CDVA improved in 14 out of 20 eyes (70%) and the UNVA in 18 out of 20 (90%) eyes. Particularly for patients receiving MIOLs, evaluation of vision-related quality of life shoud be considered [66]. Within this study patients reported a significant improvement in visual-related items of the 25-item Visual Function Questionnaire (VFQ-25) in both unilateral and bilateral MIOL groups. It was concluded that such an approach provided an improvement in near vision that is not available with standard cataract surgery.

### Uncertainty about the future

Progression of a macular disease after cataract and refractive lens exchange is a problematic issue [59], as patients might develop macular diseases years after the primary surgery. Thus, it might be questioned whether a MIOL should be disadvised in a diabetic patient who is at risk of developing macular edema or should we advise against MIOLs only in patients with a present macular edema or in all diabetics? There is no evidence that MIOLs should be disadvised in these patients.

One doubt that remains is what levels of CS should be considered as normal, given its large standard deviation in healthy subjects? Whether MIOL should be compared with age-matched phakic or pseudophakic monofocal IOL subjects? What level of reduction in CS should be considered as clinically significant? And finally, what is the threshold in CS to contraindicate MIOLs?

## Conclusions

We were unable to find evidence suggesting that patients with macular diseases should be advised against MIOLs. Several contraindications for MIOLs in patients with retinal diseases have a hypothetical character and are not evidence-based. More research is needed especially to address the effect of MIOLs on CS, visual functions, and patient activities such as orientation, mobility, and reading in various retinal pathologies.

## Electronic supplementary material

ESM 1 (DOCX 12 kb)

## References

[1] Grzybowski A, Kanclerz P (2019) Recent developments in cataract surgery. Current Concepts in Opthalmology 55–97. 10.1007/978-3-030-25389-9_3

[2] Grzybowski A, Kanclerz P (2019) Do we need day-1 postoperative follow-up after cataract surgery? Graefe's Archive for Clinical and Expiremental Opthalmology 257(5):855–61 10.1007/s00417-018-04210-0 30569320

[3] European Registry of Quality Outcomes for Cataract and Refractive Surgery. http://www.eurequo.org/. Accessed 13 Feb 2019

[4] Arden GB, Jacobson JJ (1978). A simple grating test for contrast sensitivity: preliminary results indicate value in screening for glaucoma. Invest Ophthalmol Vis Sci.

[5] Oen FT, Lim TH, Chung MP (1994). Contrast sensitivity in a large adult population. Ann Acad Med Singap.

[6] Klein BE, Klein R, Jensen SC (1996). Visual sensitivity and age-related eye diseases. The beaver dam eye study. Ophthalmic Epidemiol.

[7] Karatepe AS, Köse S, Eğrilmez S (2017). Factors affecting contrast sensitivity in healthy individuals: a pilot study. Turk J Ophthalmol.

[8] Sia DIT, Martin S, Wittert G, Casson RJ (2013). Age-related change in contrast sensitivity among Australian male adults: Florey adult male ageing study. Acta Ophthalmol.

[9] Hohberger B, Laemmer R, Adler W (2007). Measuring contrast sensitivity in normal subjects with OPTEC 6500: influence of age and glare. Graefes Arch Clin Exp Ophthalmol.

[10] Nomura H, Ando F, Niino N (2003). Age-related change in contrast sensitivity among Japanese adults. Jpn J Ophthalmol.

[11] Nio YK, Jansonius NM, Fidler V (2000). Age-related changes of defocus-specific contrast sensitivity in healthy subjects. Ophthalmic Physiol Opt.

[12] Burton KB, Owsley C, Sloane ME (1993). Aging and neural spatial contrast sensitivity: photopic vision. Vis Res.

[13] Nameda N, Kawara T, Ohzu H (1989). Human visual spatio-temporal frequency performance as a function of age. Optom Vis Sci.

[14] Tulunay-Keesey U, Ver Hoeve JN, Terkla-McGrane C (1988). Threshold and suprathreshold spatiotemporal response throughout adulthood. J Opt Soc Am A.

[15] Crassini B, Brown B, Bowman K (1988). Age-related changes in contrast sensitivity in central and peripheral retina. Perception.

[16] Sloane ME, Owsley C, Jackson CA (1988). Aging and luminance-adaptation effects on spatial contrast sensitivity. J Opt Soc Am A.

[17] Higgins KE, Jaffe MJ, Caruso RC, deMonasterio FM (1988). Spatial contrast sensitivity: effects of age, test–retest, and psychophysical method. J Opt Soc Am.

[18] Elliott DB (1987). Contrast sensitivity decline with ageing: a neural or optical phenomenon?. Ophthalmic Physiol Opt.

[19] Yates JT, Terry Yates J, Harrison JM (1987). Contrast sensitivity. Optom Vis Sci.

[20] Ross JE, Clarke DD, Bron AJ (1985). Effect of age on contrast sensitivity function: uniocular and binocular findings. Br J Ophthalmol.

[21] Morrison JD, McGrath C (1985). Assessment of the optical contributions to the age-related deterioration in vision. Q J Exp Physiol.

[22] Kline DW, Schieber F, Abusamra LC, Coyne AC (1983). Age, the eye, and the visual channels: contrast sensitivity and response speed. J Gerontol.

[23] Owsley C, Sekuler R, Siemsen D (1983). Contrast sensitivity throughout adulthood. Vis Res.

[24] McGrath C, Morrison JD (1981). The effects of age on spatial frequency perception in human subjects. Q J Exp Physiol.

[25] Sekuler R, Hutman LP, Owsley CJ (1980). Human aging and spatial vision. Science.

[26] Derefeldt G, Lennerstrand G, Lundh B (1979). Age variations in normal human contrast sensitivity. Acta Ophthalmol.

[27] Elliott D, Whitaker D, MacVeigh D (1990). Neural contribution to spatiotemporal contrast sensitivity decline in healthy ageing eyes. Vis Res.

[28] Andersen GJ (2012). Aging and vision: changes in function and performance from optics to perception. Wiley Interdiscip Rev Cogn Sci.

[29] Morrison JD, Jay JL (1993). Changes in visual function with normal ageing, cataract and intraocular lenses. Eye.

[30] Pedrotti E, Carones F, Aiello F (2018). Comparative analysis of visual outcomes with 4 intraocular lenses: monofocal, multifocal, and extended range of vision. J Cataract Refract Surg.

[31] Grzybowski A, Kanclerz P, Muzyka-Woźniak M (2019). Methods for evaluating quality of life and vision in patients undergoing lens refractive surgery. Graefes Arch Clin Exp Ophthalmol.

[32] Rosen E, Alió JL, Dick HB (2016). Efficacy and safety of multifocal intraocular lenses following cataract and refractive lens exchange: metaanalysis of peer-reviewed publications. J Cataract Refract Surg.

[33] Wang SY, Stem MS, Oren G (2017). Patient-centered and visual quality outcomes of premium cataract surgery: a systematic review. Eur J Ophthalmol.

[34] Altemir-Gomez I, Millan MS, Vega F, et al (2019) Comparison of visual and optical quality of monofocal versus multifocal intraocular lenses. Eur J Ophthalmol 1120672119827858 10.1177/1120672119827858 30739479

[35] Mencucci R, Favuzza E, Caporossi O (2018). Comparative analysis of visual outcomes, reading skills, contrast sensitivity, and patient satisfaction with two models of trifocal diffractive intraocular lenses and an extended range of vision intraocular lens. Graefes Arch Clin Exp Ophthalmol.

[36] Dyrda A, Martínez-Palmer A, Martín-Moral D (2018). Clinical results of diffractive, refractive, hybrid multifocal, and monofocal intraocular lenses. J Ophthalmol.

[37] Pedrotti E, Mastropasqua R, Bonetto J (2018). Quality of vision, patient satisfaction and long-term visual function after bilateral implantation of a low addition multifocal intraocular lens. Int Ophthalmol.

[38] Maxwell A, Holland E, Cibik L (2017). Clinical and patient-reported outcomes of bilateral implantation of a 2.5 diopter multifocal intraocular lens. J Cataract Refract Surg.

[39] Plaza-Puche AB, Alio JL, Sala E, Mojzis P (2016). Impact of low mesopic contrast sensitivity outcomes in different types of modern multifocal intraocular lenses. Eur J Ophthalmol.

[40] Labiris G, Giarmoukakis A, Patsiamanidi M (2015). Mini-monovision versus multifocal intraocular lens implantation. J Cataract Refract Surg.

[41] Gil MA, Varón C, Cardona G (2014). Comparison of far and near contrast sensitivity in patients symmetrically implanted with multifocal and monofocal IOLs. Eur J Ophthalmol.

[42] Tan N, Zheng D, Ye J (2014). Comparison of visual performance after implantation of 3 types of intraocular lenses: accommodative, multifocal, and monofocal. Eur J Ophthalmol.

[43] Yamauchi T, Tabuchi H, Takase K (2013). Comparison of visual performance of multifocal intraocular lenses with same material monofocal intraocular lenses. PLoS One.

[44] Ji J, Huang X, Fan X, Luo M (2013). Visual performance of Acrysof ReSTOR compared with a monofocal intraocular lens following implantation in cataract surgery. Exp Ther Med.

[45] Wilkins MR, Allan BD, Rubin GS (2013). Randomized trial of multifocal intraocular lenses versus monovision after bilateral cataract surgery. Ophthalmology.

[46] Zhao G, Zhang J, Zhou Y (2010). Visual function after monocular implantation of apodized diffractive multifocal or single-piece monofocal intraocular lens. J Cataract Refract Surg.

[47] Martínez Palmer A, Gómez Faiña P, España Albelda A (2008). Visual function with bilateral implantation of monofocal and multifocal intraocular lenses: a prospective, randomized, controlled clinical trial. J Refract Surg.

[48] Cillino S, Casuccio A, Di Pace F (2008). One-year outcomes with new-generation multifocal intraocular lenses. Ophthalmology.

[49] Harman FE, Maling S, Kampougeris G (2008). Comparing the 1CU accommodative, multifocal, and monofocal intraocular lenses: a randomized trial. Ophthalmology.

[50] Zeng M, Liu Y, Liu X (2007). Aberration and contrast sensitivity comparison of aspherical and monofocal and multifocal intraocular lens eyes. Clin Exp Ophthalmol.

[51] Sen HN, Sarikkola A-U, Uusitalo RJ, Laatikainen L (2004). Quality of vision after AMO array multifocal intraocular lens implantation. J Cataract Refract Surg.

[52] Montés-Micó R, España E, Bueno I (2004). Visual performance with multifocal intraocular lenses: mesopic contrast sensitivity under distance and near conditions. Ophthalmology.

[53] Leyland MD, Langan L, Goolfee F (2002). Prospective randomised double-masked trial of bilateral multifocal, bifocal or monofocal intraocular lenses. Eye.

[54] Kamlesh DS, Kaushik S (2001). Contrast sensitivity and depth of focus with aspheric multifocal versus conventional monofocal intraocular lens. Can J Ophthalmol.

[55] Haaskjold E, Allen ED, Burton RL (1998). Contrast sensitivity after implantation of diffractive bifocal and monofocal intraocular lenses. J Cataract Refract Surg.

[56] Allen ED, Burton RL, Webber SK (1996). Comparison of a diffractive bifocal and a monofocal intraocular lens. J Cataract Refract Surg.

[57] Percival SPB, Setty SS (1993). Prospectively randomized trial comparing the pseudoaccommodation of the AMO ARRAY multifocal lens and a monofocal lens. J Cataract Refract Surg.

[58] Steinert RF, Post CT, Brint SF (1992). A prospective, randomized, double-masked comparison of a zonal-progressive multifocal intraocular lens and a monofocal intraocular lens. Ophthalmology.

[59] Braga-Mele R, Chang D, Dewey S (2014). Multifocal intraocular lenses: relative indications and contraindications for implantation. J Cataract Refract Surg.

[60] de Vries NE, Webers CAB, Touwslager WRH (2011). Dissatisfaction after implantation of multifocal intraocular lenses. J Cataract Refract Surg.

[61] Alio JL, Plaza-Puche AB, Férnandez-Buenaga R (2017). Multifocal intraocular lenses: an overview. Surv Ophthalmol.

[62] Aychoua N, Junoy Montolio FG, Jansonius NM (2013). Influence of multifocal intraocular lenses on standard automated perimetry test results. JAMA Ophthalmol.

[63] Prakash G, Prakash DR, Agarwal A (2011). Predictive factor and kappa angle analysis for visual satisfactions in patients with multifocal IOL implantation. Eye.

[64] Kamath GG, Prasad S, Danson A, Phillips RP (2000). Visual outcome with the array multifocal intraocular lens in patients with concurrent eye disease. J Cataract Refract Surg.

[65] Gayton JL, Mackool RJ, Ernest PH (2012). Implantation of multifocal intraocular lenses using a magnification strategy in cataractous eyes with age-related macular degeneration. J Cataract Refract Surg.

[66] Grzybowski A, Kanclerz P, Muzyka-Woźniak M (2019) Methods for evaluating quality of life and vision in patients undergoing lens refractive surgery. Graefe's Archive for Clinical and Experimental Ophthalmology 257:1091–99 10.1007/s00417-019-04270-w 30824995

