# Supplementary material for: Multifocal intraocular lenses and retinal diseases
Source: Graefes Arch Clin Exp Ophthalmol. 2020 Jan 18;258(4):805–13. doi: 10.1007/s00417-020-04603-0 (PMC7575463; doi:10.1007/s00417-020-04603-0)
Supplement: Supplementary file 1 — (DOCX 12 kb) [file 417_2020_4603_MOESM1_ESM.docx]

**Appendix 1. Search strategy**

Literature searches of the PubMed and Web of Science databases were conducted in June 30, 2019; the search strategies are as follows. Specific limited update searches were conducted after June 30, 2019.

**A.1. PubMed Search (Publication Date 1/1/1900–6/30/2019)**

(((“multifocal intraocular lens”[Title]) OR (“cataract surgery”[Title]) OR (“cataract extraction”[Title]) OR (“lens exchange”[Title])) AND ((“diabetic retinopathy”[Title]) OR (“age-related macular degeneration”[Title]) OR (“contrast sensitivity”[Title]))). 172 references.

**A.2. Web of Science Search (Publication Date 1/1/1900–6/30/2019)**

((TI=(multifocal intraocular lens) OR TI=(cataract surgery) OR TI=(cataract extraction) OR TI=(lens exchange)) AND (TI=(diabetic retinopathy) OR TI=(age-related macular degeneration) OR TI=(contrast sensitivity))) Indexes=SCI-EXPANDED, SSCI, A&HCI, CPCI-S, CPCI-SSH, BKCI-S, BKCI-SSH, ESCI, CCR-EXPANDED, IC Timespan=All years. 235 references.
